# Editorial for Special Issue “Maternal, Fetal and Neonatal Health”

**DOI:** 10.3390/healthcare11172461

**Published:** 2023-09-04

**Authors:** Mohamed E. Abdel-Latif

**Affiliations:** 1 Department of Neonatology, Centenary Hospital for Women and Children, Canberra Hospital, Garran, Canberra, ACT 2606, Australia; abdel-latif.mohamed@act.gov.au; Tel.: +61-2-6174-7565; 2 Department of Public Health, College of Science Health and Engineering, La Trobe University, Bundoora, Melbourne, VIC 3083, Australia; 3 Discipline of Neonatology, School of Medicine and Psychology, College of Health and Medicine, Australian National University, Acton, Canberra, ACT 2601, Australia

The maternal, foetal, and neonatal health field has witnessed remarkable advancements in recent years, driven by cutting-edge research and innovative technologies. Our journal proudly presents a special thematic series of articles focused on critical perinatal and neonatal medicine topics to spotlight these developments. This Special Issue aims to bridge the gap between research and clinical practice, fostering a deeper understanding of new possibilities and encouraging discussions within the perinatal and neonatal medicine community.

We explore groundbreaking research on this issue that unveils new perinatal and neonatal medicine possibilities. We aim to inspire further exploration, collaboration, and innovation by presenting these breakthroughs.

The thematic series covers a wide range of topics, and consists of the following papers:

- “Mobilising the World Café Method for Adequate Development of Non-Technical Skills of Midwives” [1];
- “Evaluation of Midwives’ Practises on Herpetic Infections during Pregnancy” [2];
- “Physical Violence during Pregnancy and Its Implications” [3];
- “Experience in Perinatal Exposure to HIV Infection” [4];
- “Pregnant Women’s Satisfaction with Model of Care Initiative” [5];
- “The Birth Plan Experience” [6];
- “The Current Concept of Paternal Bonding” [7];
- “Queens and Wet Nurses—Indispensable Women in the Dynasty of the Sun King [8];
- The Mediation Effect of Coping Strategies and Pregnancy Complicated by Hypertension” [9];
- “Development and Validation of a Risk Scoring Tool for Bronchopulmonary Dysplasia in Preterm Infants” [10];
- “Vibroacoustic Study in the Neonatal Ward” [11].

This Special Issue is a testament to the progress made in perinatal and neonatal medicine. By presenting the latest research, translating findings into actionable recommendations, and presenting the newest research from a broad range of institutions from around the world, this Special Issue strives to improve outcomes and quality of life for mothers and their newborns around the world.
